# Supplementary material for: Noninvasive Models to Assess Liver Inflammation and Fibrosis in Chronic HBV Infected Patients with Normal or Mildly Elevated Alanine Transaminase Levels: Which One Is Most Suitable?
Source: Diagnostics (Basel). 2024 Feb 20;14(5):456. doi: 10.3390/diagnostics14050456 (PMC10930419; doi:10.3390/diagnostics14050456)
Supplement: Supplementary file 1 [file diagnostics-14-00456-s001.zip › diagnostics-2859237-supplementary.pdf]

Table S1. Calculations of the 55 noninvasive models.

Noninvasive Models	developed cohort	Calculations
**AA index**	CHB	=-9.164+0.114×AFP+0.236×APTT
**AAR**	CHC	=AST(IU/L) / ALT(IU/L)
**AARPRI**	CHC or CHB	=AAR/(PLT/150)
**ABA**	CHC	=1.5+[age×0.065]+[bilirubin total(mg/dL)×1.85]−[albumin (g/dL)×1.65]
**AGAP**	CHB	=[AST (IU/L) × GGT(IU/L)] × [Age (years) / PLT^2]
**AGPR**	CHB	=[ALP (U/L) + GGT (U/L)]/PLT count (10^9/L)
**ALRI**	CHC	=AST/Lymphocytes
**AP index**	CHC	**APind = Age + Platelet counts**
		**Age** (yr): <30=0, 30~39=1, 40~49=2, 50~59=3, 60~69=4, ≥70=5
		**Platelet counts** (10^9/L): ≥225=0, 200~224=1, 175~199=2, 150~174=3, 125~149=4, <125=5
**APRI**	CHC	=AST (ULN) ×100 / PLT (10^9/L)
**APGA**	CHB	=1.44+0.1490×log(GGT)+0.3308×log(AST)-0.5846×log(PLT)+0.1148×log(AFP+1)
**APPCI**	CHB	=-28.89+1.157×logAFP(ng/ml)+30.284×logPT(s)-0.018×PLT(10^{11}/L)-0.023×CP(mg/L)
**APPR**	CHB	(AKP, IU/ml)/(PLT, 10^9 /l).
**APRG**	CHB	1/(1+e^{-A})
		A=-6.091+ALP×0.015-PLT×0.01+RDW-SD×0.118+GLO×0.081
**ATPI model**	CHB	=0.054×AST (g/L) + 0.09×TBil (umol/L) – 0.008× PLT (10^9 /L) – 0.366
**CDS**	CHC	CDS = PLT + ALT/AST + INR
		**PLT** (10^9/L): ≥340=0, 280~339=1, 220~279=2, 160~219=3, 100~159=4, 40~99=5, <40=6
		**ALT/AST**: >1.7=0, 1.2~1.7=1, 0.6~1.19 =2, <0.6=3
		**INR**: <1.1=0, 1.1~1.4=1, >1.4=2
**Doha score**	CHC	=8.5-0.2 ×ALB (g/dl) + 0.01 ×AST (IU/L) - 0.02× PLT (10^9/L)
**eLIFT**	CHB	eLIFT is the sum of age, gender, GGT, AST, platelet count, and prothrombin time
		Age (years): <40=0, ⩾40=3;
		Gender: female=0, male=1;
		AST (IU/L): <35=0, 35－69=2, ⩾70=4;
		GGT (IU/L): <35=0, 35~89=1, ⩾90=2;
		Platelet count (109 /L):⩾250=0, 170~249=1, <170=4;
		Prothrombin time (%):⩾97=0, 84~96=2, <84=4;
**FCI**	CHC	=ALP (IU/L) ×TBil (mg/dl) / ALB (g/dl) / PLT (10^9/L)

**FI**	CHC	=8.0 - 0.01 × PLT (10^9/L) - ALB (g/dl)
**FIB-4**	CHC	=Age (yr) ×AST (IU/L) / (PLT (10^9/L) × ALT (IU/L)$^{1/2}$)
**mFIB-4**	CHB or CHC	=10×Age×AST/(PLT×ALT)
**FIB-5**	CHC	=albumin (g/L) × 0.3+ platelet count (10^9/L) × 0.05−ALP (IU/L)×0.014+AST/ALT ratio×6 + 14
**FIB-6**	CHC	A nomogram based on age,AST,ALT,ALP,ALB,PLT, which could be found at **http://fib6.elriah.info/**
**FibroQ**	CHC	=10 ×Age (yr) × AST (IU/L) × PT INR / (ALT (IU/L) × PLT (10^9/L))
**Forns**	CHC	=7.811-3.131×ln(PLT)+0.781×ln(GGT)+3.467×ln(Age)-0.014×CHOL(mg/dl)
**Fibro- α**	CHC	=1.35+ [AFP ×0.009584]+ [(AST)/(ALT) × 0.243] -(platelet count × 0.001624)
**GAPI**	CHB	=-5+0.05×GGT+0.03×Age-0.005×Platelet(10^9/L)+5×INR
**Gao-2**	CHB	=0.049×Age+2.858×ln(AST)-0.352×$\log_{10}$(HBV-DNA IU/ml×5)-0.02×PLT+0.213
**Gao-1**	CHB	=5.956×$\log_{10}$(AST)-2.612×$\log_{10}$(HBsAg/1000)-0.016×PLT-0.15×ALB+9.544
**GqHBsR**	CHB	=GGT to HBsAg ratio
**GP**	CHB	=GLB (g/dl) × 100 / PLT (10^9/L)
**GPR**	CHB	=GGT (IU/L) / GGT ULN / PLT (10^9/L) ×100
**GUCI**	CHC	=AST (ULN) ×PT INR ×100 / PLT (10^9/L)
**HBeAg(+)model**	CHB	=5.956× $\log_{10}$ (AST)-2.612× $\log_{10}$(HBsAg) - 0.016× PLT (× 10^9/L) - 0.15× ALB(g/L) + 9.544
**HB-F**	CHB	=0.018 × [Age(yr)] + 1.085 ×[AST/ALT] - 0.009 ×[Platelet (10^3/mm^3)] + 0.449 × [Prothrombin time prolongation (s)]
**HGM-1**	CHC	1/(1+e^{-A})<br>A=-1.971-0.0121×Platelet(10^9/L)+0.026×AST(UI/dL)+0.033×Glucose(mg/dL)
**HGM-2**	CHC	1/(1+e^{-A})<br>A=-6.175+0.010×Platelet(10^9/L)+4.8×INR-0.01×ALP(UI/dL)+0.007×AST(UI/dL)
**IT model**	CHB	=9.39-2.6×log(HBsAg)+0.05×GGT
**INPR**	CHB	=INR/PLT×100
**King's score**	CHC	=Age (yr) × AST (IU/L) × INR / PLT (10^9/L)
**Lok index**	CHC	Log odds (predicting cirrhosis) = -5.56-0.0089×Platelet count (10^9 /L)+1.26×AST/ALT ratio+5.27×INR<br>Predicted probability = exp (log odds) / [1 + exp (log odds)]
**Logit(Y)**	CHB	=-0.09+1.15×AST+0.07×GGT-0.03×PLT-0.65×$\log_{10}$(HBsAg)
**Mehdi's model**	CHB	=10+0.771×$\log_{10}$(HBVDNA[copies/mL])+3.828×$\log_{10}$ (ALP/ULN)-1.066×albumin(g/dL)-0.011× (platelets/1000 μL).
**NIKEI**	NAFLD	LogitP=ln(P/1-P)=-24.214+0.225×age+0.056×AST (IU/L)+5.044×AST/ALT ratio+3.631×total bilirubin (mg/dl)
**NLR**	CHC, CHB, NAFLD	=Neutrophil/Lymphocyte
**PAPAS**	CHB	=0.0255+0.0031×Age+0.1483×log(ALP)+0.004×log(AST)+0.0908×log(AFP+1)-0.028×log(PLT)
**PGA**	CHC	The sum of PTA, GGT and Apo-A1 score

		PTA: ⩾80=0; 70~79=1; 60~69=2; 50~59=3; <50=4;
		GGT: <20=0; 20~49=1; 50~99=2; 100~199=3; ⩾200=4;
		Apo-A1: ⩾200=0; 175~199=1; 150~174=2; 125~149=3; <125=4
**PNALT**	CHB	$=-2.632+0.93\times ALT+0.258\times \log_{10}HBV\text{-}DNA-0.995\times sex$ (male=1, female=2).
**RPR**	CHB	=RDW%/ Platelet Ratio
**RLR**	PBC	=RDW%/ Lymphocyte Ratio
**S index**	CHB	$=1000\times GGT(IU/L)/(PLT(10^9/L)\times ALB(g/L)^2$
**Virahep-C model**	CHC	$y = -5.17 + 0.20\times Race + 0.07\times Age\ (yr) + 1.19\ \ln\ (AST\ [IU/L]) -1.76\ \ln\ (PLT\ [10^9/L]) + 1.38\ \ln\ (ALP\ [IU/L])$
		race: Caucasian Americans=1, African Americans=0
		Predicted probability = 1 / (exp (-y) + 1)
**Wang I**	CHB	$A=0.153-0.015\times PLT(10^9/L)+0.154\times AST(IU/L)+0.071\times GGT(IU/L)-0.226\times \ln\ (HBVDNA(copies/ml))$
		Wang $I=10\times e^A/(1+e^A)$
**Wang II**	CHB	$Y=\exp(-3.755+0.742\times Sex-0.015\times Age-0.01\times PLT-0.209\times TC+0.021\times GGT+0.379\times PT)$
		male sex = 1, female sex = 0, PLT in 10^9 /L, TC in mmol/L, GGT in IU/L and PT in seconds.
		Wang II=Y/(1+Y)
**XIE-model**	CHB	$=9.871+0.018\times ALT+0.033\times AST-0.182\times ALB+0.016\times ALP+0.012\times GGT-0.057\times PTA$

Table S2. Area under the ROC curve(AUROC) of models for liver fibrosis and necro-inflammation with different levels of ALT under 2 ULN (ULN=40U/L)

Models	ALT<1×ULN (n=434)		ALT≥1×ULN (n=165)		ALT<1×ULN (n=434)		ALT≥1×ULN (n=165)	
	S≥2（n=126）	S≥3（n=42）	S≥2（n=62）	S≥3（n=22）	G≥2（n=118）	G≥3（n=29）	G≥2（n=73）	G≥3（n=21）
AA index	0.595	0.688	0.605	0.667	0.645	0.684	0.591	0.648
AAR	0.542	0.605	0.671	**0.701**	0.545	0.651	0.593	**0.713**
AARPRI	0.624	0.698	**0.711**	**0.764**	0.614	**0.740**	0.699	**0.805**
ABA	0.538	0.631	0.643	0.687	0.643	0.687	0.666	**0.703**
AGAP	**0.719**	**0.800**	0.681	**0.770**	**0.718**	**0.858**	**0.715**	**0.834**
AGPR	0.699	**0.783**	0.688	**0.735**	0.689	**0.808**	**0.701**	**0.808**
ALRI	0.565	0.632	0.595	0.668	0.564	0.689	0.606	0.650
APGA	**0.766**	**0.840**	**0.707**	**0.797**	**0.774**	**0.872**	**0.739**	**0.859**
APind	0.607	0.680	0.672	0.640	0.602	0.693	**0.717**	**0.703**
APRI (ULN=40U/L)	**0.701**	**0.767**	0.653	**0.752**	0.690	**0.827**	**0.719**	**0.812**

APPCI	**0.728**	**0.788**	**0.705**	**0.779**	**0.728**	**0.726**	0.686	**0.749**
APPR	0.666	**0.723**	0.674	**0.714**	0.659	**0.743**	0.691	**0.776**
APRG	0.680	**0.801**	0.696	**0.765**	**0.714**	**0.846**	**0.715**	**0.824**
ATPI model	0.647	**0.737**	0.637	**0.738**	0.612	**0.759**	0.669	**0.773**
CDS	0.638	**0.720**	0.675	**0.721**	0.632	**0.718**	**0.701**	**0.773**
Doha score	0.651	**0.726**	0.666	**0.738**	0.638	**0.735**	**0.739**	**0.798**
eLIFT	0.668	**0.765**	0.680	0.699	0.665	**0.784**	**0.705**	**0.742**
FCI	0.664	**0.744**	0.664	**0.702**	0.628	**0.740**	0.674	**0.730**
FI	0.652	**0.760**	0.677	**0.763**	0.661	**0.814**	**0.765**	**0.828**
FIB-4	0.641	**0.714**	**0.709**	**0.755**	0.659	**0.757**	**0.729**	**0.797**
mFIB-4	0.605	0.681	**0.719**	**0.749**	0.621	**0.717**	**0.715**	**0.782**
FIB-5	0.383	0.345	0.362	0.330	0.390	0.319	0.251	0.267
FIB-6(ULN=40)	0.623	**0.706**	0.693	**0.787**	0.662	**0.804**	**0.762**	**0.837**
FibroQ	0.619	**0.700**	**0.728**	**0.755**	0.629	**0.725**	**0.720**	**0.785**
Forns	0.634	**0.748**	0.664	0.682	0.613	**0.740**	0.692	**0.736**
Fibro-α	0.677	**0.747**	**0.718**	**0.811**	0.667	**0.776**	**0.731**	**0.852**
GAPI	**0.734**	**0.821**	0.666	**0.750**	**0.709**	**0.838**	0.658	**0.803**
Gao-2	0.674	**0.742**	0.672	**0.759**	0.674	**0.776**	0.688	**0.802**
Gao-1	0.620	0.666	0.666	**0.722**	0.647	**0.731**	0.637	**0.739**
GqHBsR	0.655	**0.701**	0.617	0.625	0.647	**0.745**	0.550	0.671
GP	0.652	**0.743**	0.644	**0.728**	0.667	**0.803**	0.678	**0.766**
GPR	**0.716**	**0.792**	0.639	**0.725**	0.699	**0.826**	0.646	**0.790**
GUCI	**0.716**	**0.786**	0.668	**0.766**	0.688	**0.830**	**0.724**	**0.820**
HBeAg(+)model	0.620	0.666	0.666	**0.722**	0.647	**0.731**	0.637	**0.739**
HB-F	0.662	**0.755**	0.698	**0.754**	0.654	**0.750**	**0.715**	**0.773**
HGM-1	0.645	**0.730**	0.629	**0.734**	0.632	**0.767**	0.693	**0.794**
HGM-2	0.450	0.436	0.412	0.414	0.441	0.402	0.370	0.359
IT model	0.554	0.578	0.603	0.569	0.566	**0.573**	0.495	0.561
INPR	0.666	**0.746**	0.669	**0.713**	0.635	**0.729**	**0.717**	**0.754**

King's score	0.683	**0.756**	0.692	**0.752**	0.683	**0.798**	**0.733**	**0.798**
Lok index	0.675	**0.762**	0.692	**0.758**	0.648	**0.757**	**0.715**	**0.776**
Logit(Y)	**0.713**	**0.764**	0.611	**0.726**	**0.715**	**0.862**	0.664	**0.769**
Mehdi's model	0.595	0.636	0.556	0.559	0.634	**0.701**	0.639	0.606
NIKEI	0.549	0.620	0.659	**0.701**	0.569	0.665	0.630	**0.702**
NLR	0.468	0.525	0.439	0.471	0.440	0.470	0.442	0.455
PAPAS	0.623	0.675	0.664	0.652	0.677	0.695	0.651	0.688
PGA	0.570	0.575	0.460	0.515	0.532	0.622	0.503	0.556
PNALT	0.600	0.582	0.445	0.550	0.600	0.639	0.559-	0.604-
RPR	0.634	**0.714**	**0.703**	**0.757**	0.699	0.611	**0.750**	**0.825**
RLR	0.463	0.518	0.571	0.629	0.483	0.460	0.558	0.588
S index	**0.726**	**0.812**	0.650	**0.749**	**0.715**	**0.869**	0.677	**0.816**
Virahep-C model	0.636	0.692	0.671	**0.722**	0.668	**0.747**	**0.708**	**0.778**
Wang I	**0.711**	**0.790**	0.657	**0.784**	**0.702**	**0.864**	0.649	**0.829**
Wang II	**0.705**	**0.785**	0.659	0.683	0.637	**0.752**	**0.707**	**0.780**
XIE-model	**0.721**	**0.805**	0.676	**0.799**	**0.722**	**0.863**	**0.746**	**0.847**

TableS3. Grade distribution of the 55 noninvasive models for diagnosing liver fibrosis and necroinflammation under different ALT

Grades	Noninvasive models or indexes ALT<1 ULN group	Noninvasive models or indexes in ALT 1- 2 ULN group
A	APGA	
B	AGAP AGPR APRI APPCI APRG FI GAPI GPR GUCI Logit(Y) S index Wang I Wang II XIE-model	RPR APGA[#] FI FIB-6 AARPRI AGAP APRI APRG FIB-4 FibroQ GUCI XIE-model AGPR mFIB-4 Fibro-α GAPI Gao-2 HB-F King's score Lok index Wang I
C	AA index AAR AARPRI ABA ALRI AP index APPR ATPI model CDS Doha score FCI mFIB-4	APPCI GPR Logit(Y) Mehdi’s model S index AA index AAR ABA ALRI AP index

	FIB-5 FIB-6 FibroQ Fibro-α eLIFT Forns<br>FIB-4 Gao-1 Gao-2 HBeAg(+)model<br>King's score GqHBsR GP HB-F HGM-1 HGM-2<br>IT model INPR Lok index Mehdi’s model<br>NLR NIKEI PAPAS PGA PNALT<br>RPR RLR Virahep-C model	APPR ATPI model CDS Doha score eLIFT FCI<br>mFIB-4 FIB-5 Forns Gao-1 GqHBsR GP HBeAg(+)model<br>HGM-1 HGM-2 IT model INPR<br>NLR NIKEI PAPAS PGA PNALT RPR RLR<br>Virahep-C model Wang II

Note: # the highest sum scores of models in group was 8, and the score of APGA was 7.

Abbreviations: AASLD, American Association for the Study of Liver Diseases; AIH, autoimmune hepatitis; ALT, alanine transaminase; AST, aspartate transaminase; ALP, alkaline phosphatase; ALB, albumin; AF, advanced fibrosis; AFP, alpha-fetoprotein; AUROC, area under the receiver operating characteristic curve; BMI, body mass index; CHB, chronic hepatitis B; CHC, chronic hepatitis C; CHE, cholinesterase; CI, confidence interval; CLD, chronic liver disease; CPA, collagen proportionate area; CT, computerized tomography; CDS, cirrhosis discriminant score; DNA, deoxyribonucleic acid; EASL, European Association for the Study of the Liver; FI, fibrosis index; FCI, fibrosis cirrhosis index; GLB, globulin; γ-GGT or GGT, gamma-glutamyl transpeptidase; HBV, hepatitis B virus; HBsAg, hepatitis B surface antigen; HBeAg, hepatitis B e antigen;HCV, hepatitis C virus; HCC, hepatocellular carcinoma; HCT, hematocrit; HDV, hepatitis D virus; HIV, human immunodeficiency virus; Hp, haptoglobin; HA, hyaluronic acid; INR, international normalized ratio; IA, immune active; IT, immune tolerant ; liver biopsy; LC, liver cirrhosis; MRI, magnetic resonance-diffusion tension imaging; MMP-1, matrix metalloproteinase-1; PT, prothrombin time; PTA, Prothrombin activity; PLT, platelet; PIIINP, procollagen III N-terminal peptide; PBC, primary biliary cholangitis; PSC, primary sclerosing cholangitis; ALD, alcoholic liver disease; NAFLD, nonalcoholic fatty liver disease; RBC, red blood cell; RDW-SD, red blood cell distribution width-SD; RDW-CV, red blood cell distribution width-CV; ROC, receiver operating characteristic curves; SF, significant fibrosis; sCD163, soluble CD163; TBIL, total bilirubin; TE, transient elastography; TCHO, total Cholesterol; TBA, total bile acid; ULN, upper limit of normal; α2-MG, α2-Macroglobulin; 2D-SWE, Two-dimensional shear wave elastography Se, sensitivity; Sp, specificity; PPV, positive predictive value; NPV, negative predictive value;AAR, aspartate alanine aminotransferase ratio; APRI, aspartate aminotransferase to platelet ratio index; GUCI, Goteborg University Cirrhosis Index score; HGM-1 and HGM -2, Hospital Gregorio Marañón; LR, likelihood ratio;APGA index, AST/platelet/GGT/AFP index.
